# The impact of epidural analgesia on delivery mode in Robson class 1 women: a retrospective cohort study

**DOI:** 10.1016/j.xagr.2023.100207

**Published:** 2023-04-05

**Authors:** Fedora Ambrosetti, Giovanni Grandi, Elisabetta Petrella, Veronica Sampogna, Lara Donno, Laura Rinaldi, Anna Maria Ghirardini, Fabio Facchinetti

**Affiliations:** 1 Department of Medical and Surgical Sciences for Mother, Child, and Adult, University of Modena and Reggio Emilia, Azienda Ospedaliero Universitaria Policlinico, Modena, Italy (Dr Ambrosetti, XX Grandi, XX Petrella, XX Sampogna, and XX Facchinetti); 2 Anesthesia and Intensive Care Unit, University of Modena and Reggio Emilia, Azienda Ospedaliero Universitaria Policlinico, Modena, Italy (XX Donno, XX Rinaldi, and XX Ghirardini); 3 Anesthesia Unit, Ospedale di Sassuolo, Sassuolo, Italy (XX Ghirardini)

**Keywords:** cesarean delivery, cervical dilation, epidural analgesia, mode of delivery, neonatal outcomes, nulliparous women, obstetrical anesthesia, Robson classification, vaginal delivery

## Abstract

**BACKGROUND:**

The use of epidural analgesia represents the gold standard for pain management during labor, but the influence of the use of epidural analgesia on delivery mode is not fully understood.

**OBJECTIVE:**

This study aimed to analyze the impact of epidural analgesia on the delivery mode, namely, cesarean delivery, vaginal delivery, and operative vaginal delivery rates, in Robson class 1 women.

**STUDY DESIGN:**

A retrospective cohort study was conducted on all Robson class 1 women who delivered from January 1, 2019, to December 31, 2019, in the University Hospital of Modena. The primary outcome was the delivery mode (cesarean delivery, vaginal delivery, and operative vaginal delivery rates), and the secondary outcomes were maternal, anesthesiologic, and neonatal effects of epidural analgesia (duration of labor, duration of the second stage of labor, Apgar score, and neonatal intensive care unit admission).

**RESULTS:**

A total of 744 women were included in the final analysis, of which 198 (26.6%) underwent epidural analgesia on request and 546 (73.4%) did not. In women with and without epidural analgesia, the cesarean delivery rate was 8.1% vs 7%, the vaginal delivery rate was 79.3% vs 81.1%, and the operative vaginal delivery rate was 12.6% vs 11.9%, respectively. A significant increase in both the first stage of labor (66.3±38.5 vs 43.8±38.8 minutes; *P*<.0001) and total duration of labor (328.0±206.7 vs 201.7±168.3 minutes; *P*<.0001) was found in women receiving epidural analgesia. No change was recorded in the second stage of labor. A shorter duration of labor was observed (*P*<.0001) when epidural analgesia was started earlier (dilation: 2–4 cm vs >4 cm). No significant difference in Apgar score and neonatal intensive care unit admission was found.

**CONCLUSION:**

The use of epidural analgesia was not associated with an increased risk of cesarean delivery or operative vaginal delivery in Robson class 1 women. Further investigations are needed to evaluate its impact on the duration of labor, namely the duration of the first stage of labor, and on the possible advantages of starting epidural analgesia at an early stage.

**AJOG MFM at a Glance**

**Why was this study conducted?**

The use of epidural analgesia (EA) represents the gold standard procedure for adequate pain management. Moreover, avoiding primary cesarean deliveries seems to be of utmost importance.

**Key findings**

The delivery mode in Robson class 1 women was not negatively affected by the use of EA.

**What does this add to what is known?**

Analyzing the impact of EA in nulliparous women who enter labor spontaneously provides a unique perspective for a satisfying and secure pain management.

## Introduction

The use of epidural analgesia (EA) represents the gold standard for pain management during labor.^1^ However, controversies are rising on the routine use of EA.^2^ Attention has been focused on the impact that EA might have on the progress of labor and the mode of delivery, namely, the incidence of cesarean delivery (CD) and operative vaginal delivery (OVD).^3^ Moreover, the maternal (ie, postpartum hemorrhage [PPH] and vaginoperineal severe injuries) and neonatal (ie, 5-minute Apgar score, need for resuscitation, and neonatal intensive care unit [NICU] admission) outcomes of women choosing EA have been closely analyzed as well.^4^

New techniques have been developed to achieve satisfying pain control and to avoid interfering with the length of labor.^5^ The latest Cochrane review stated that the use of EA is not associated with an increase in CDs, but its use could be linked to a higher rate of OVDs compared with the use of opioids, even though there is a bias regarding the dosage of EA.^6^

As the number of CDs has dramatically increased during the last decades,^7^^,^^8^ the World Health Organization (WHO) report^9^ released in 2018 focused on the importance of avoiding primary CD, mainly in term nulliparous women with single cephalic newborns, reducing the risks associated with delivery in subsequent pregnancies. Previously, in 2015, the WHO proposed the Robson classification system as a global standard for assessing, monitoring, and comparing CD rates within healthcare facilities over time and among facilities.^10^ Thus, Robson Class 1 women appeared crucial in setting CD rates.

This study aimed to assess the impact of EA on the delivery mode of singleton cephalic nulliparous women at the term of pregnancy who enter labor spontaneously (Robson class 1) in our institution 1 year before the COVID-19 pandemic.

## Materials and Methods

We conducted a retrospective cohort study collecting data on deliveries that occurred from January 1, 2019, to December 31, 2019, at Policlinico Hospital of Modena, Mother Infant Department. The study was approved by the Ethics Committee of Area Vasta Emilia Nord (protocol number: 772/2020/OSS*/AOUMO).

Patients who met the criteria for Robson class 1 were eligible for enrollment. Patients who had CD performed before entering labor; those who had induction of labor for pathologic conditions (any diabetes mellitus treated with insulin, gestational hypertension, amniotic fluid anomalies, and fetal growth anomalies), for postterm pregnancy (41 3/7 weeks of gestation),^11^ or for premature rupture of membranes >24 hours; and those with incomplete medical records were excluded.

Because of the COVID-19 pandemic, we decided to investigate deliveries occurring during 2019 to minimize possible confounding factors emerging since March 2020.^12^

Data were obtained from an electronic database, and they were anonymized before analysis. All patients included in the study gave their written consent for the anonymous use of their clinical data for research purposes.

The population was analyzed according to demographic characteristics (age, education, and ethnicity), gestational weight gain (GWG), body mass index (BMI) before pregnancy, ethnicity, need for in vitro fertilization, pregnancy parameters (days of gestation at delivery and delivery after 41 weeks of gestation), and obstetrical issues (gestational diabetes mellitus [GDM] or gestational hypertension, all entering labor spontaneously). Women in our population could access the hospital at any degree of progression of labor.

The primary outcome was defined by the delivery mode, categorized as vaginal delivery (VD), CD, and (OVD). All members of the obstetrical staff agreed on the indications for CD and OVD. Among these indications, intrapartum cardiotocography and labor progression were interpreted following the international guidelines,^13^^,^^14^ and OVD was performed using a vacuum extraction technique at the middle or low level,^15^ according to local protocols.

Maternal secondary outcomes were blood loss and PPH, duration of labor (also divided into first and second stages of labor), need for augmentation with oxytocin and the rate of intrapartum fever, meconium-stained fluid, and degrees of perineal tears. Blood loss was expressed in milliliters and was evaluated by adding the blood collected inside a retroplacental pouch or in a surgical aspirator to the blood loss estimated by weighing the gauzes. PPH was defined as a blood loss exceeding 500 mL in VD or OVD and a blood loss exceeding 1000 mL in CD.^16^^,^^17^

The duration of labor was calculated considering the time (expressed in minutes) between the starting visit of the partogram^18^ and delivery. The duration of the second stage of labor was calculated considering the time (expressed in minutes) from the beginning of the pushing efforts to delivery. Women undergoing CD were excluded from this data analysis for possible bias interference. Moreover, a second stage of labor of >2 hours was considered among the secondary otucomes.^19^

Cervical dilation at the opening of the partogram is usually ≥4 cm, as obstetrical visits are not always conducted at regular time intervals in our clinical practice. To achieve a comprehensive analysis of the first stage of labor, we applied an index showing the cervical dilation rate calculated by dividing the centimeters needed to achieve full dilation, as registered on the delivery medical record, by the time in hours (dilation cm/h).

Augmentation with oxytocin was practiced in women with infrequent contraction by infusion of 5 IU of oxytocin in 500 mL of lactated Ringer's solution following the regimens prescribed by international guidelines.^20^

Neonatal secondary outcomes included birthweight, Apgar score of <7^21^ at 1 and 5 minutes, abnormal cord blood gas analysis^22^ (arterial pH of ≤7 or base deficit of >12 mmol/L), and need for NICU admission.

According to the local protocol of the obstetrical anesthesia team and the most recent meta-analysis,^23^^,^^24^ the epidural catheter is placed at L3 to L4 or L4 to L5 interspace, and the EA is provided at low doses (sufentanil and levobupivacaine 0.0625%) at intermittent boluses aiming to a walking analgesia rather than to the lowest pain score. During labor, pain is evaluated using the numeric rating scale from 0 to 10, corresponding to the absence of pain and to the worst pain felt. Women willing to require EA during labor attend a brief set of lessons held by our anesthesiology team during the third trimester of pregnancy and provide informed consent to the procedure.

Anesthesiologic secondary outcomes were divided by early vs late start of EA administered by bolus injections through the epidural catheter.^24^ An early start of EA was defined as the administration of the first bolus of the drug before the cervix dilates to 4 cm. In contrast, we defined a late start of EA as the administration of the first bolus of the drug when the cervix dilates to >4 cm.^25^ Data were compared for delivery mode, duration of labor, duration of the second stage of labor, and dilation rate (cm/h).

### Statistical analysis

The characteristics of women undergoing EA and the control group were analyzed and compared. In the descriptive analysis, the continuous variables were summarized by the mean and standard deviation (SD), and the categorical variables were reported as absolute and percentage values. The distribution of continuous covariates by the group was compared using the 1-way analysis of variance. The comparison of categorical variables between groups was performed using the chi-square test or Fisher exact test, when appropriate. Multivariate logistic regression analyses were used to investigate risk factors associated with delivery mode and PPH occurrence concerning the use of EA, adjusting for confounding, such as age, BMI, ethnicity, neonatal birthweight, labor duration, and need for augmentation: candidate variables were included if significant on univariate analysis or clinically relevant. Statistical analysis was performed using the statistical package StatView (version 5.01.98; SAS Institute Inc, Cary, NC). Correlations were considered to be significant at *P*<.05. The results of continuous data are expressed as mean±SD.

## Results

### Baseline features

A total of 744 Robson class 1 women were included in the final analysis with a mean age of 31.2±5.2 years. Their features are reported in Table 1 according to EA (n=198 [27%]) and non-EA (n=546 [73%]). The formers are significantly older (*P*=.0005) with a higher GWG (*P*=.002), despite similar prepregnancy BMI (*P*=.74). Moreover, in this group, there were more women of White origin (*P*<.0001). No women had pre-GDM or chronic hypertension. The prevalence of GDM or gestational hypertension was similar among the 2 groups, all entering labor spontaneously before programmed induction of labor.

**Table 1:** Features of women according to analgesic choice

Variables	EA (n=198)	No EA (n=546)	*P* value
Age (y)	31.2±4.3	29.8±5.3	.0005
Prepregnancy BMI (kg/cm^2^)	22.4±3.6	22.5±3.6	.74
BMI			.11
<18.5	12 (6.1)	51 (9.3)	
18.5–24.9	153 (77.3)	383 (70.1)	
25.0–29.9	22 (11.1)	89 (16.3)	
>30.0	11 (5.5)	23 (4.2)	
GWG at 36 wk (kg)	12.1±3.5	11.0±4.2	.002
White ethnicity	184 (92.9)	422 (77.3)	<.0001
IVF	9 (4.5)	18 (3.3)	.62
Days of gestation at delivery	280.0±9.0	279.3±12.5	.497
Delivery—after 41 0/7 wk of gestation	24 (12.1)	75 (13.7)	.57
Gestational diabetes mellitus without medical treatment	8 (4.0)	36 (6.6)	.19
Gestational hypertension	2 (1.0)	4 (0.7)	.71

Data are presented as mean±standard deviation or number (percentage), unless otherwise indicated.

*BMI*, body mass index; *EA*, epidural analgesia; *GWG*, gestational weight gain; *IVF*, in vitro fertilization.

### Primary outcome

The delivery mode was not significantly different in patients with and without EA in terms of CD rate (8.1% vs 7%), VD rate (79.3% vs 81.1%), and OVD rate (12.6% vs 11.9%) (*P*=.83) (Table 2). Moreover, this result was confirmed by a logistic regression model, including maternal age, BMI, ethnicity, the use of EA, and neonatal birthweight as independent variables: CD occurrence was not related to the use of EA (CC, 1.2; 95% confidence interval [CI], 0.6–2.4; *P*=.68).

**Table 2:** Primary and secondary outcomes

Variables	EA (n=198)	No EA (n=546)	*P* value
Delivery mode			.83
CD	16 (8.1)	38 (7.0)	
VD	157 (79.3)	443 (81.1)	
OVD	25 (12.6)	65 (11.9)	
Duration of labor (min)	328.0±206.7	201.7±168.3	<.0001
Progression of the first stage of labor (cm/h)	0.90±0.50	1.37±1.20	<.0001
Second stage of labor			
Duration (min)	50.7±2.4	46.9±1.44	.17
>2 h (%)	11 (6.0)	23 (4.5)	.42
Augmentation	111 (56.1)	102 (18.7)	<.0001
CD at full dilation	3 (1.5)	14 (2.6)	.397
Reasons for CD			.39
CTG category II or III	6 (37.5)	18 (47.4)	
Prolonged labor	6 (37.5)	16 (42.1)	
Both reasons	4 (25.0)	4 (10.5)	
Blood loss (mL)			
All deliveries	462.4±28.6	393.6±13.0	.013
VD + OVD	463.7±30.7	386.5±13.4	.008
CD	446.9±60.8	488.2±50.4	.64
Postpartum hemorrhage (VD + OVD) (>500 mL)	66 (33.3)	138 (25.3)	<.001
Intrapartum fever of >38.5°C	7 (3.5)	5 (0.9)	.01
Meconium-stained amniotic fluid	41 (20.7)	110 (20.1)	.87
Episiotomy	19 (10.4)	65 (12.8)	.40
Vaginoperineal tears—grade 1 or no injury	121 (66.5)	338 (66.5)	—
Vaginoperineal tears—grade 2	56 (30.7)	156 (30.7)	—
Vaginoperineal tears—grade 3	5 (2.7)	12 (2.3)	—
Vaginoperineal tears—grade 4	0 (0)	2 (0.3)	—

Data are presented as number (percentage) or mean±standard deviation, unless otherwise indicated.

C*D*, cesarean delivery; *CTG*, cardiotocography; EA, epidural analgesia; OVD, operative vaginal delivery; VD, vaginal delivery.

### Secondary outcomes

A significant increase was found in total duration of labor (328.0±206.7 vs 201.7±168.3 minutes; *P*<.0001) in women with EA, especially for the first stage of labor, expressed as dilation rate (cm/h) in women undergoing EA compared with women not undergoing EA (0.9±0.5 vs 1.37±1.2 cm/h; *P*<.0001) (Table 2). In contrast, the duration of the second stage of labor, either as total duration (50.7±2.4 vs 46.9±1.4; *P*=.17) or considering a prolonged period of more than 2 hours of pushing (6% in cases vs 4.5% in controls; *P*=.42) was unaffected.^26^ In addition, we did not find any difference in CD performed at dilation ≥10 cm in the 2 groups (1.5% [EA] vs 2.6% [no EA]; *P*=.397) and among the indications for CD (*P*=.39).

A significant need for augmentation with oxytocin was found in the EA group (56.1% vs 18.7%; *P*<.0001).

Moreover, if EA was started early (2–4 cm), both the duration of labor (270.0±179.0 vs 420±212.8 minutes; *P*<.0001) and the dilation rate (1.1±0.66 vs 0.7±0.4 cm/h; *P*<.0001) were significantly reduced with a similar duration of the second stage of labor (51.8±34.8 vs 49.4±31.4 minutes; *P*=.63) compared with EA started at a dilation of >4 cm (Table 3).

**Table 3:** Comparisons of early vs late EA

Variables	Early EA (2–4 cm) (n=122)	Late EA (>4 cm) (n=76)	*P* value
Method of delivery			.96
CD	10 (8.0)	6 (7.8)	
OVD	16 (13.0)	9 (11.8)	
Duration of labor (min)	270.0±179.0	420.0±212.8	<.0001
Progression of the first stage of labor (cm/h)	1.10±0.66	0.70±0.40	<.0001
Duration of the second stage of labor (min)	51.8±34.8	49.4±31.4	.63

Data are presented as number (percentage) or mean±standard deviation, unless otherwise indicated.

C*D*, cesarean delivery; EA, epidural analgesia; OVD, operative vaginal delivery.

We found a significant increase in blood loss in patients undergoing EA, either considering all deliveries (462.4±28.6 vs 393.6±13.0 mL; *P*=.013) or vaginal deliveries only (VD+OVD) (463.7 ±30.7 vs 386.5±13.4 mL; *P*=.008). Moreover, this evidence was found in the PPH rate after VD and OVD as well (66.0% vs 25.3%; *P*<.001). No statistical difference was found for blood loss after CD, neither for total blood loss (*P*=.64) nor for PPH (*P*=.247) (Table 2). In a multivariate model, including age, BMI, ethnicity (White vs non-White), neonatal birthweight, the use of EA, need for augmentation, and labor duration as independent variables, the occurrence of PPH was independently related (r=0.07) only to neonatal weight (CR, 2.9; 95% CI, 1.8–4.7; *P*<.0001) and ethnicity (CR, 1.8; 95% CI, 1.1–2.9; *P*=.01). The use of EA did not enter the equation (CR, 1.4; 95% CI, 0.9–2.1; *P*=.1).

We noted a significant increase in intrapartum fever of >38.5°C in women undergoing EA (3.5% vs 0.9%; *P*=.01), although we noted similar rates of meconium-stained amniotic fluid (20.7% vs 20.1%; *P*=.87).

Considering perineal tears, no difference was noted in any extent of lacerations and in the episiotomy rate (10.4% vs 12.8%; *P*=.40).

We recorded 2 shoulder dystocia cases that occurred in women undergoing OVD in the EA group.

### Neonatal outcomes

A higher birthweight was found in women undergoing EA (3350.6±26.7 vs 3277.3±16.7 g; *P*=.02). However, we did not note any significant differences in Apgar score at 1 and 5 minutes, abnormal cord blood gas analysis, base excess, or NICU admission rates between the 2 newborn groups (Table 4).

**Table 4:** Neonatal outcomes

Variables	EA (n=198)	No EA (n=546)	*P* value
Apgar score of <7			
1 min	16 (8.1)	46 (8.4)	.88
5 min	3 (1.5)	10 (1.8)	.77
Cord blood gas analysis			
Umbilical artery pH of <7.10	6/190 (3.2)	22/462 (4.7)	.36
BE of ≥12	7/190 (3.6)	21/462 (4.5)	.62
NICU admission	5 (2.5)	11 (2.0)	.67

Data are presented as number (percentage), unless otherwise indicated. Concerning cord blood gas analysis, data are presented as number/total number (percentage).

BE, base excess; EA, epidural analgesia; NICU, neonatal intensive care unit.

## Discussion

### Principal findings

We conducted a retrospective cohort study aimed at evaluating the impact of EA on the delivery mode in women in Robson class I. No difference between groups was found for CD, VD, or OVD. Moreover, there was no change noted between early and late EA.

### Results

These data are in contrast with our regional report of birth certificates (“Certificato di Assistenza al Parto” 2018)^27^ where an increased risk of CD and OVD was associated with EA. The latest research studies showed conflicting results in this respect. In cohort studies,^4^^,^^28^^,^^29^ EA was correlated to a higher rate of OVD and longer first and second stages of labor with an increased likelihood of receiving oxytocin infusion and developing an intrapartum fever. In contrast, in a Chinese study^30^ released in 2017 and in a meta-analysis published in the same year,^31^ no correlation was found between EA and the mode of delivery. In a 10-year cohort study performed in the Netherlands, no causative effect was found between EA and OVD.^32^

### Clinical implications

In our study, EA was significantly associated with a longer duration of labor mainly because of an increase in the first stage of labor without affecting the length of the second stage of labor. Variable effects have been noticed regarding the effects of EA on the first stage of labor.^33^ In nulliparous women, labor might take longer than expected based on the Friedman curve,^34^^,^^35^ regardless of the use of EA. A longer first stage of labor does not correspond in our study to an increase in the rate of CD performed for prolonged labor, in both populations. Longer dilating periods seem to be associated with heterogeneous effects of EA on uterine activity, as proposed by Lim et al,^36^ even in a specialized setting such as ours.

Regarding the effects on the second stage of labor, there is a large consent for allowing a longer time for women undergoing EA and delaying the pushing efforts until the rectal pressure is well perceived by the woman.^19^^,^^37^ If the fetal status is reassuring, then potentially unnecessary interventions could be avoided.^38^ We implemented such updated pieces of evidence in our daily practice. Moreover, our study was conducted in a small town in Italy, and women could be admitted at various degrees of cervical dilation, as long as they lived not far from the hospital. We did not have enough information to discuss the possible effects of this practice, although they might seem encouraging.^39^

Concerning EA performed in the early stage of labor, recent studies have failed to link an early initiation at a cervical dilation of <4 cm to an increase in CD.^36^ The Cochrane review released in 2014^37^ stated that EA could be safely administered at maternal request. Moreover, early EA could result in faster labor, as shown by Wong et al^38^ and as confirmed in our population.

Women undergoing EA presented an increased likelihood of receiving augmentation with oxytocin. Despite being controversial, the association between EA and oxytocin need has been outlined in various studies,^40^, ^41^, ^42^ and it seems to be correlated either with the effect of EA on uterine contractility or with underlying risk factors for labor dystocia, such as fetal macrosomia, malpresentation, or inefficient uterine activity. However, not enough data were registered in our archives to establish the main reason for this medical choice for the multiple uses of oxytocin in labor.

Unexpectedly, EA was correlated to a higher blood loss at delivery and to a higher risk of PPH after VDs, whereas undergoing a CD would not imply a higher risk of bleeding. Considering that PPH has been associated with various risk factors,^43^^,^^44^ our multivariate analysis found correlations only between neonatal birthweight and maternal ethnicity (White vs non-White).

A higher birthweight was found in the EA group, although this result was not related to an increased rate of episiotomy performed or to a worse extent of vaginoperineal tears. Myrick et al^45^ confirmed our findings in their recent research. Concerning the 2 shoulder dystocia cases recorded, both were without any neonatal consequence, neonatal birthweights exceeded 4000 g, and none of their mothers had diabetes mellitus. Although the shoulder dystocia rate in women undergoing EA is similar to literature findings,^46^^,^^47^ our data seem inconsistent, considering the size of our population.

The association between the use of EA and intrapartum fever has not yet been completely understood.^4^ Sterile inflammation and activation of inflammatory response most probably play a crucial role,^48^^,^^49^ without compromising fetal wellness. Our data confirmed a higher incidence of intrapartum fever in women undergoing EA with similar rates in meconium-stained amniotic fluid and NICU admissions.

Our pediatric outcomes did not show any worsening of neonatal well-being parameters in our population, as reported in a recent cohort study.^29^ Although they appear in contrast with Ravelli et al^50^ research, not enough data were available to further inquiry our results.

### Research implications

As a result of our research, further studies on the impact of EA on the durations of the first and second stages of labor are needed to analyze the incidence of maternal fever during EA, the differences in maternal blood loss, and, eventually, the advantages of starting EA at an early stage of labor.

### Strengths and limitations

The main limitations of this retrospective study are related to the size of the population involved and the short period analyzed. The strengths of our study are represented by a novel approach to studying the use of EA, as we decided to focus our attention specifically on Robson class 1 women only and on the advantages of an early EA. This eventually raised questions on the impact of EA on the induction of labor.

## Conclusions

EA in Robson class 1 women and EA started early in labor were not associated with a higher rate of CD or instrumental deliveries in our population. Women undergoing EA experienced longer labor, lost more blood, and delivered heavier babies, although EA was not related to any worsening neither in the neonatal conditions nor in the extent of vaginoperineal tears. Comprehensive management of labor is needed to allow enough time to complete every stage of labor for women who choose EA, although further investigations are needed to analyze the role of each confounding factor and the impact of EA on neonatal outcomes.

The important result of the absence of additional risks of CD or OVD in nulliparous women who enter labor spontaneously indicates a good point in understanding how EA works. Further multicenter studies are needed to safely offer EA to all women in labor.
